# Deleterious mutations in *ALDH1L2* suggest a novel cause for neuro-ichthyotic syndrome

**DOI:** 10.1038/s41525-019-0092-9

**Published:** 2019-07-23

**Authors:** Catherine Sarret, Zahra Ashkavand, Evan Paules, Imen Dorboz, Peter Pediaditakis, Susan Sumner, Eléonore Eymard-Pierre, Christine Francannet, Natalia I. Krupenko, Odile Boespflug-Tanguy, Sergey A. Krupenko

**Affiliations:** 1 IGCNC, Institut Pascal, UMR CNRS-UCA-SIGMA, Aubière, France; 2 Department of Clinical Genetics and Medical Cytogenetics, Centre Hospitalier Universitaire de Clermont-Ferrand, Clermont-Ferrand, France; 3 Nutrition Research Institute, University of North Carolina, Chapel Hill, NC USA; 4 Department of Nutrition, University of North Carolina, Chapel Hill, NC USA; 5 INSERM UMR1141, DHU PROTECT, PARIS-DIDEROT, University Sorbonne Paris-Cite, Paris, France; 6 Department of Child Neurology and Metabolic Disorders, LEUKOFRANCE, Hôpital Robert Debré, Assistance Publique-Hôpitaux de Paris, Paris, France

**Keywords:** Diseases, Metabolic disorders

## Abstract

Neuro-ichthyotic syndromes are a group of rare genetic diseases mainly associated with perturbations in lipid metabolism, intracellular vesicle trafficking, or glycoprotein synthesis. Here, we report a patient with a neuro-ichthyotic syndrome associated with deleterious mutations in the *ALDH1L2* (aldehyde dehydrogenase 1 family member L2) gene encoding for mitochondrial 10-formyltetrahydrofolate dehydrogenase. Using fibroblast culture established from the ALDH1L2-deficient patient, we demonstrated that the enzyme loss impaired mitochondrial function affecting both mitochondrial morphology and the pool of metabolites relevant to β-oxidation of fatty acids. Cells lacking the enzyme had distorted mitochondria, accumulated acylcarnitine derivatives and Krebs cycle intermediates, and had lower ATP and increased ADP/AMP indicative of a low energy index. Re-expression of functional ALDH1L2 enzyme in deficient cells restored the mitochondrial morphology and the metabolic profile of fibroblasts from healthy individuals. Our study underscores the role of ALDH1L2 in the maintenance of mitochondrial integrity and energy balance of the cell, and suggests the loss of the enzyme as the cause of neuro-cutaneous disease.

## Introduction

Neuro-ichthyotic syndromes are a group of rare genetic diseases mainly associated with perturbations in lipid metabolism, intracellular vesicle trafficking, or glycoprotein synthesis.^1^ Congenital dry and scaly skin and progressive neurological symptoms are hallmarks of this group of diseases. Sjögren–Larsson syndrome (SLS: MIM#270200) is one of the most recognized neuro-ichthyotic syndromes characterized by congenital ichthyosis, leukoencephalopathy, intellectual disability, and spastic di- or tetraplegia.^2–4^ In 95% of patients it is caused by mutations of the *ALDH3A2* gene which encodes for the fatty aldehyde dehydrogenase (FALDH), a microsomal enzyme that oxidizes long-chain aldehydes to fatty acids.^2,5,6^ SLS patients without mutations in the *ALDH3A2* gene have also been identified, leaving the cause of the symptoms unknown.^7^ Here, we report a patient with a congenital neuro-ichthyotic syndrome but atypical phenotype displaying dysmorphic features, and abnormalities on MRI and MR (^1^H-MRS) spectroscopy in the absence of *ALDH3A2* gene mutations and no spastic paraplegia to suggest classic SLS. The diagnosis of Coffin–Lowry syndrome, made after identification of a deleterious frameshift mutation in the *RPS6KA3* gene,^8,9^ does not explain all features of the patient. We provide evidence that the neuro-ichthyotic syndrome in this case is associated with the loss of expression of the *ALDH1L2* gene, which encodes a mitochondrial folate enzyme.

## Results

### Patient’s developmental history and morphological features

**Fig. 1:**
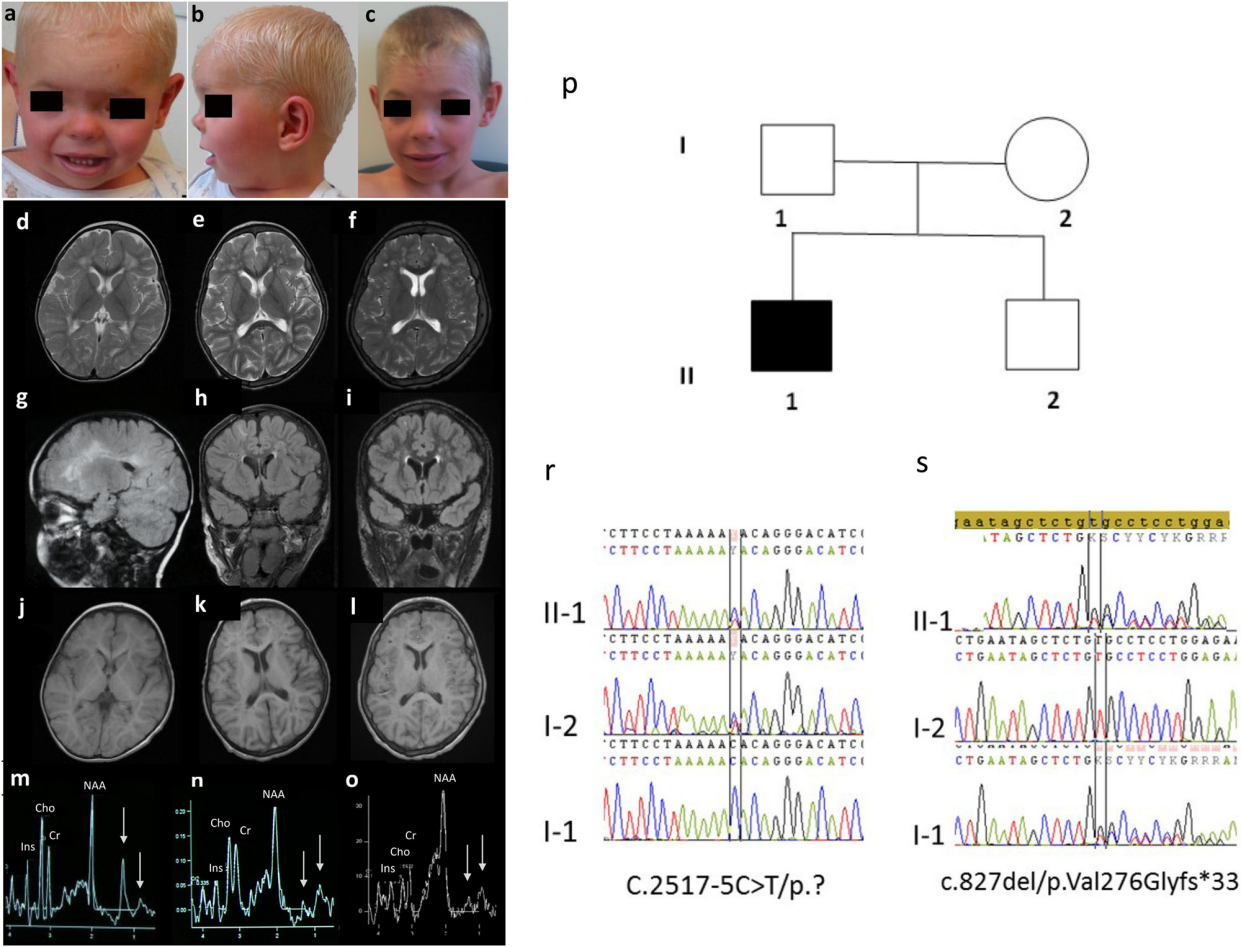
Particularities of the patient phenotype. Patient at the age of 3 years **a**, **b** and 14 years **c** presented with a facial dysmorphism with epicanthus, hypertelorism, broad nasal root, anteverted nares, long philtrum, thin upper lip. The written consent for publication of these photos was obtained from child’s parents. Cerebral MRI shows diffuse hypomyelination at the age of 2 years with white matter appearing respectively in hypersignal on T2-weighted sequences, in hypersignal on FLAIR sequences and in normosignal on T1-weighted sequences **d**, **g**, **j**. Progressive myelination and dilatation and coalescing of Virchow–Robin spaces at the age of 6 **e**, **h**, **k** and 14 years **f**, **i**, **l**. ^1^H-MRS in the corona radiata for classical SLS patients shows a typical major peak at 1.3 ppm and a smaller peak at 0.9 ppm (**m**, arrows). ^1^H-MRS for our patient reveals a similar pattern with two peaks at 1.3 and 0.9 ppm (arrows) at the age of 2 **n** and 6 years **o**. However, the peak at 1.3 ppm appears smaller than the peak at 0.9 ppm in our patient. We observed no decrease in N-acetyl-aspartate (NAA)/creatine (Cr) ratio, or in choline (Cho) peak suggesting normal maintenance of neuronal and myelin content but a small increase of inositol peak that may be due to some astrocytic stress. Family pedigrees **p** and patient’s genotype **r**, **s**

The patient (male) presented at birth with hypotonia, abnormally thick fingers and toes, and ichthyosis. Pruritis and facial dysmorphism were apparent since the age of 11 months (Fig. 1). He had normal statural, ponderal, and head circumference growth. Motor acquisitions during the 1st year were severely delayed with sitting acquired at 11 months and independent walking at 3 years. At 14 years, the patient was able to speak using efficient isolated words and had good communication skills. He was unable to read and presented hyperactivity and attention deficit increasing over time. To date, the patient had normal walking and has not developed neurological signs including spastic paraplegia or ataxia. Morphological studies of skeleton, abdomen, and heart were normal. Electroencephalogram showed diffuse moderate bradyrythmia. Somatosensory evoked potentials revealed prolonged latencies on the four limbs and motor evoked potentials showed the lack of cortical response. MRI at 1.5 T demonstrated early diffuse hypomyelination with coalescing and dilated Virchow–Robin spaces. ^1^H-MRS reveals two abnormal lipid peaks in the white matter persistent at short and long echo times (other peaks were normal, except a small increase of inositol) (Fig. 1). Positions of these peaks in our patient correspond to positions of the characteristic peaks in classic SLS but the overall profile was different. Specifically, in SLS the 1.3 ppm peak is more prominent while the 0.9 ppm peak is smaller (Fig. 1m). In our patient, the peaks were still evident at 6 years of age, the finding which in association with the leukodystrophy initially suggested the SLS diagnosis. Of note, in SLS patients both peaks are permanent through age while in our patient these peaks decreased over time and were not detected after 8 years of age (Supplementary Fig. 1).

### Mutations identified by whole exome sequencing

Sequencing of the *ALDH3A2* exons, exon/intron junctions, and the full-length cDNA did not reveal any mutations in this gene in our patient. The following mutations were found by the whole exome analysis: (1) a de novo hemizygous mutation (c.263dup, p.Ser89Leufs*4) in the *RPS6KA3* gene on the X chromosome,^10,11^ a deleterious frameshift mutation not carried by the patient’s mother. This finding confirmed that the patient was affected by a Coffin–Lowry syndrome^8^ and partially explained the patient’s features but did not explain the congenital pruritic ichthyosis, MRI or ^1^H-MRS features typical of SLS. (2) This patient also has compound heterozygous mutations in the *ALDH1L2* gene, which encodes for a mitochondrial 10-formyltetrahydrofolate dehydrogenase.^12^ Discovered mutations, one intronic near splice site (c.2517-5C>T) and one frame shift (c.827del/p.Val276Glyfs*33; rs770401066, dbSNP NCBI) were not present in the homozygous state in ExAC or gnomAD. The patient’s asymptomatic parents and brother were heterozygous for one of these mutations. Segregation analysis revealed that the father harbors the Val276Glyfs*33 frameshift mutation, while the mother harbors the c.2517-5C>T intronic mutation. The presence of a mutant mRNA resulting from the frame shift was confirmed in patient’s fibroblasts by the direct sequencing. The mutated sequence predicts a truncated protein of 307 aa, including the 22 aa mitochondrial leader sequence, 253 aa of the N-terminal folate-binding domain/hydrolase catalytic center,^13^ and the 32 aa random peptide with no identity to known proteins resulted from the frameshift (Supplementary Fig. 2). Such truncated proteins are usually not folded properly,^13^ and apparently are rapidly degraded. Indeed, the truncated protein was not detected in the patient’s fibroblasts. Deficiency of the *ALDH1L2* gene has not been reported and the overall consequences of the enzyme loss for the cell are not clear. We examined fibroblasts from this patient, his parents and healthy unrelated individual, and present evidence that the loss of ALDH1L2 impairs the mitochondrial function and is the likely cause of a new neuro-ichthyotic syndrome.

### Characterization of patient’s fibroblasts

**Fig. 2:**
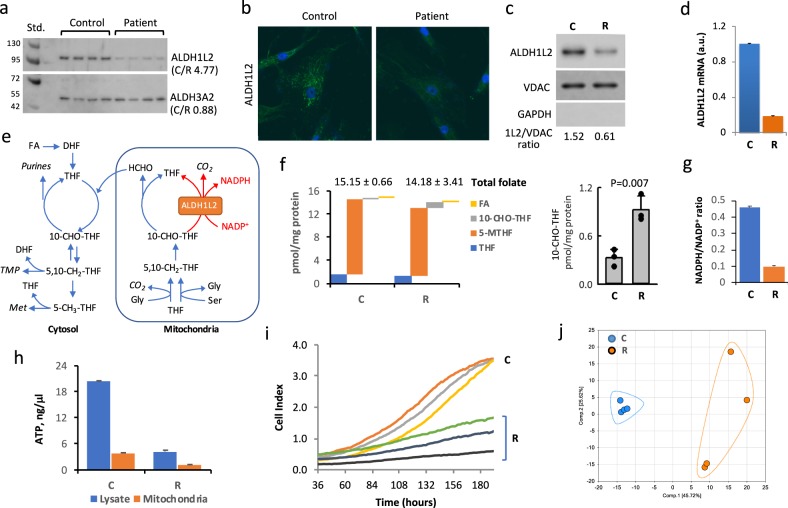
Difference between fibroblasts from the patient (R cells) and fibroblasts from healthy individual (C cells). **a**–**c** R cells have much lower levels of ALDH1L2 protein as evaluated by Western blot assay (**a**, ratios of averaged band intensities are indicated; statistics is shown in Supplementary Fig. 3) and confocal microscopy **b** in cells or by Western blot assay in isolated mitochondria (**c**). In panel **a**, samples were from different plates (biological replicates) with 20 μg of the total protein loaded per well. **d** Levels of ALDH1L2 mRNA are lower in R cells (mean ± SE of three biological replicates). **e** Cytosolic and mitochondrial folate pathways. **f** Levels of folate coenzymes (FA, folic acid; THF, tetrahydrofolate; 5-MTHF, 5-methyl-THF; 10-CHO-THF, 10-formyl-THF) in C and R cells (only 10-CHO-THF was noticeably and significantly different between the two cell lines). For each cell type mean ± SE of three independent experiments (each done in quadruplicate) is shown (3 biological replicates each includes 4 technical replicates). **g** Ratio of NADPH/NADP^+^ in C and R cells (mean ± SE of four biological replicates). **h** Levels of ATP in C and R cells (mean ± SE of four biological replicates). For panels **d**, **g**, **h**, *p* values were below 0.001 (Student’s *t*-test) for the comparison of R and C cells (detailed statistical analysis for these panels is shown in Supplementary Figs. 4 and 6–8). **i** Proliferation rate of C and R cells measured in real-time (xCelligence); samples with three densities of cells were monitored for each cell type. In each case, experiments were done in duplicate with automated averaging of data points. **j** PCA for metabolites (475 total) measured in C and R cells (*n* = 4; samples are biological replicates)

**Fig. 3:**
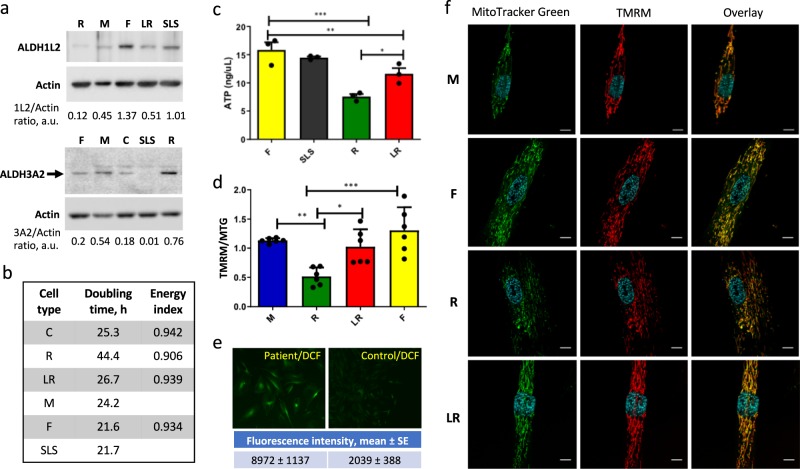
Patient fibroblasts have lower ATP and energy index, and altered mitochondrial morphology in comparison with fibroblasts from parents or SLS patient. **a** Western blots assays of ALDH1L2 and ALDH3A2 in fibroblasts isolated from the patient (R), both parents (mother, M; father, F) and from an SLS patient (SLS). LR denotes patient’s fibroblasts transduced for ALDH1L2 expression. Numbers on the bottom of each panel indicate band intensity (arbitrary units) for ALDH1L2 and ALDH3A2 relative to the intensity of corresponding actin band. **b** Doubling time and energy index of different fibroblast cultures (cell labeling as in panel **a**). **c** ATP levels measured by a colorimetric assay in different fibroblast cultures. Three different samples (biological replicates) were used in this experiment; for each sample, 4 measurements (technical replicates) were performed and the average of these measurements were used to calculate mean ± SE. **d** TMRM (tetramethylrhodamine) to MitoTracker Green ratio in different fibroblasts. Six samples (biological replicates) were analyzed for each cell type. **e** Levels of ROS evaluated by confocal microscopy after DCF (2′,7′-dichlorodihydrofluorescein diacetate) staining in patient (R cells) and control (C cells) fibroblasts (values were calculated from the analysis of 10 cells for each cell type; laser power was kept uniform for all measurements). **f** Confocal images (108×) of different fibroblast cultures (as in panel a). Live cells were stained with Hoechst (nucleus staining, *light-blue*), MitoTracker Green (mitochondrial staining, *green*), or TMRM (mitochondrial staining, *red*); scale bars, 10 µM. For panels **c**, **d**, *p < 0.05; **p < 0.01; ***p < 0.001

Compared to fibroblasts from a healthy individual (control, C cells), patient’s fibroblasts (R cells) have barely detectable ALDH1L2 protein (Fig. 2a–c and Supplementary Fig. 3). Levels of *ALDH1L2* mRNA were also significantly lower in patient’s cells (Fig. 2d and Supplementary Fig. 4). Since one of the alleles of the *ALDH1L2* gene in the patient has mutation near the splice site, we attributed the decrease in the mRNA level to the impaired transcription caused by the mutation. Indeed, splice site mutations are known to cause loss of gene expression.^14^ Levels of the ALDH3A2 protein were not different between the two cell lines (Fig. 2a and Supplementary Fig. 3), an indication that FALDH deficiency was not the primary cause of the patient’s symptoms. The ALDH1L2 enzyme catalyzes the conversion of 10-formyl-THF to THF and CO_2_ simultaneously producing NADPH from NADP^+^ (Fig. 2e).^12,15,16^ Therefore, the ALDH1L2 activity is likely to affect folate metabolism but the extent of the enzyme contribution to the maintenance of reduced folate pools is not clear. The total folate levels were not significantly different between patient’s (R) and control (C) fibroblasts (Fig. 2f), only 10-formyl-THF was noticeably and significantly different between two fibroblast cultures (Fig. 2f). ALDH1L1, the cytosolic homolog of ALDH1L2 and a major user of 10-formyl-THF^17,18^ was not present in either fibroblast culture (Supplementary Fig. 5). Therefore, the three-fold increase of this folate upon the ALDH1L2 loss (Fig. 2f) indicates that the enzyme is a major user of 10-formyl-THF. The ratio of NADPH/NADP^+^, metabolites also involved in ALDH1L2 catalysis, was more than four-fold lower in patient versus control fibroblasts (Fig. 2g and Supplementary Fig. 6), supporting the role of ALDH1L2 as the main source of NADPH generation.^19^ Furthermore, patient’s fibroblasts have much lower ATP levels in mitochondria as well as in whole cells (Fig. 2h and Supplementary Figs. 7 and 8) with the ATP/ADP ratio indicating a very low energy status in patient’s fibroblasts (Fig. 3b, d). Another characteristic feature of ALDH1L2-deficient fibroblasts is a decreased proliferation rate (Fig. 2i), which was not responsive to the increase of folate in media (10 μM leucovorin or 20 μM folic acid). In fact, the metabolomics analysis demonstrated differences between the patient and control fibroblasts beyond folate metabolism (Fig. 2j) with statistically significant (*p* < 0.05) differences for 250 out of 475 assigned metabolites.

### Comparison of our patient’s fibroblasts with fibroblasts derived from parents

Fibroblast cultures generated from both parents (mother, M, heterozygous for the intronic mutation; father, F, heterozygous for the mutation causing a premature stop codon) showed ALDH1L2 protein expression though its levels were noticeably higher in father’s cells (Fig. 3a). Both cell lines have similar levels of ALDH3A2 proteins comparable with those of patient’s fibroblasts (Fig. 3a), and both demonstrated a much faster proliferation rate than R cells as indicated by the doubling time (Fig. 3b). ATP levels in the father’s cells were remarkably higher than in R cells (Fig. 3c). Furthermore, mitochondria from R cells have lower membrane potential (Fig. 3d) and showed increased levels of reactive oxygen species (Fig. 3e). Correspondingly, metabolomic analysis has shown increased levels (fold-change > 2; *p* < 0.05) of several oxidative stress biomarkers such as methionine sulfoxide, 5-oxoproline, and ophthalmate, in patient’s cells (Supplementary Data File 1). Confocal microscopy has shown differences in mitochondrial morphology in patient’s fibroblasts with the appearance of rounded isolated mitochondria, which were not seen in healthy control or father’s fibroblasts (Fig. 3f). The morphology of mitochondria in mother’s fibroblasts was similar to the morphology of the control and father’s fibroblasts though mother’s cells were smaller in size compared to fibroblasts from other individuals (Fig. 3f). These cells as well have normal doubling time (Fig. 3b) and mitochondrial membrane potential (Fig. 3d).

**Fig. 4:**
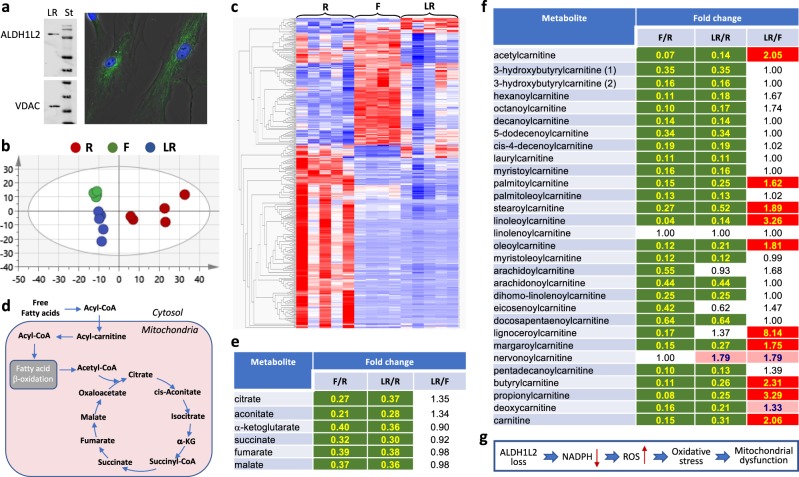
Metabolomic analysis of R, F, and LR fibroblasts. **a** Lentivirus-based expression of ALDH1L2 in LR fibroblasts restores levels of the enzyme seen in control or parent’s cells (Western blot assay of isolated mitochondria and confocal image of fibroblasts stained with ALDH1L2-specific antibody, *St* indicates lane with molecular weight standards, VDAC is shown as mitochondrial marker; *green* fluorescence indicates ALDH1L2; nuclei were co-stained with DAPI). **b** PCA (principal component analysis, performed with SIMCA Version 15.0.2, Sartorius Stedim Data Analytics AB, Umeå, Sweden) of metabolomic data (total of 516 metabolites) for R, F, and LR fibroblasts (*n* = 5 biological replicates in each case). **c** Heat map representation of the metabolite comparison between R, F, and LR cells (performed with Qlucore Omics Explorer v.3.4 software, Qlucore, Lund, Sweden; data were filtered by *p* value ≤ 0.05). **d** Schematic depicting the TCA cycle and its connection to carnitine pathway. **e**, **f** Levels of Krebs cycle metabolites and carnitine and most acylcarnitine derivatives are similar in F and LR cells but compared to R cells are much lower in both cell lines. Statistically significant differences (*n* = 5) are highlighted in *green* (*p* < 0.05, decreased metabolites), *red* (*p* < 0.05, increased metabolites), or *light red* (*p* < 0.1, increased metabolites). **g** Proposed mechanism for the effect of the ALDH1L2 loss

Electron microscopy further confirmed altered mitochondrial morphology in R cells compared to father’s cells (Supplementary Fig. 9). In contrast to mitochondria of father’s cells, which are filamentous as commonly seen in cultured fibroblasts,^20^ mitochondria of R cells appear to be shorter and distorted. Another noticeable feature of R cells was the presence of large vesicles not seen in F cells (Supplementary Fig. 9). We suggest that these alterations are linked to the metabolic effects caused by the ALDH1L2 deficiency. Thus, significant differences in metabolic profile in R cells were associated with amino acid, nucleotide, and lipid pathways (Fig. 4 and Supplementary Data File 1). Of note, accumulation of all common amino acids is indicative of decreased protein biosynthesis,^21^ which is in line with decreased proliferation and low energy status in R cells. Strong changes in the lipid profiles were seen in R cells with the most dramatic increase of acylcarnitine metabolites (Fig. 4) and the reduction of mono- and diglycerides as well as all classes of phospholipids (Supplementary Data File 1).

### Restoration of biochemical properties by expression of wild-type ALDH1L2

Re-expression of ALDH1L2 (via viral transduction) in patient fibroblasts (LR cells, Fig. 4a) restored the morphological features of father’s cells, including re-appearance of filamentous mitochondria (Fig. 3f) and disappearance of large vesicles (Supplementary Fig. 9); decreased the doubling time (Fig. 3b); increased levels of ATP (Fig. 3c) and mitochondrial membrane potential (Fig. 3d) thus improving the energy status of these cells and making them more similar to father’s fibroblasts. Overall, the metabotype of patient cells with restored ALDH1L2 expression was shifted towards the metabotype of father cells (Fig. 4b, c) indicating that the metabotype of patient cells is associated with the loss of ALDH1L2. Specifically, Krebs cycle intermediates and acyl carnitines were similar in LR and F fibroblasts (Fig. 4d–f). These data indicate that the ALDH1L2 loss affects fatty acid metabolism. In fact, several reports implicated the enzyme in β-oxidation and fatty acid metabolism^22–24^ though underlying mechanisms are not clear. Alternatively, alterations in fatty acid metabolism could be a cellular response to oxidative stress associated with the ALDH1L2 loss.^25^ The phenotype rescue by transduction of ALDH1L2 indicates that the metabolic changes and mitochondrial dysfunction were not caused by Coffin–Lowry syndrome.

### Comparison of our patient’s fibroblast with fibroblasts derived from an SLS patient

We have also compared our patient’s fibroblasts with the fibroblast culture from a patient with classical SLS caused by the loss of ALDH3A2 enzyme due to a homozygous mutation (c.471+1delG in intron 3) associated with the splicing abnormality of the *ALDH3A2* gene. Levels of ALDH1L2 appeared normal in the SLS patient and were similar to the protein level in father’s fibroblasts (Fig. 3a). These cells have a standard doubling time and ATP levels similar to father’s cells characterized in this study (Fig. 3b, d). Confocal microscopy has shown that SLS fibroblasts have a typical mitochondrial morphology (Supplementary Fig. 10a) distinct from that observed in R cells (Fig. 3f). The metabotype of SLS fibroblasts was different from the metabotype of fibroblasts from our patient as well as metabotypes of healthy control or asymptomatic parents of our patient (Supplementary Figs. 10b, 10c, and 11; and Supplementary Data File 1). These differences suggest that our patient has a distinct biochemical basis for SLS-like symptoms.

## Discussion

Patient reported here displays characteristics leading to the diagnosis of SLS-like neuro-ichthyotic syndrome. Since this patient does not have mutations in the *ALDH3A2* gene, the cause of the disease remained unclear until exome sequencing revealed inherited mutations in the *ALDH1L2* gene. The patient also has a deleterious frameshift mutation in the *RPS6KA3* gene, which encodes for ribosomal S6 kinase (RSK2), a growth factor-regulated serine/threonine kinase and a member of the RAS-MAPK signaling pathway.^10,11^ Mutations in this gene are associated with Coffin–Lowry syndrome, a rare X-linked genetic disorder characterized by intellectual disability, tapering fingers, cranio-facial and skeletal abnormalities.^8^ Affected males were also reported to have cardiac problems, obesity, short stature, microcephaly, dental abnormalities, deafness, visual problems, hypotonia, hyperlaxity, behavioral troubles, epilepsy, sleep apneas, or drop attacks.^26,27^ Brain MRI can show cerebral atrophy, hypoplasia of the corpus callosum or the cerebellar vermis, ventricular dilatation or asymmetry, dilatation of the Virchow–Robin spaces leading to periventricular white matter cystic lesions, and constricted foramen magnum.^28–31^

Recognizing Coffin–Lowry syndrome in very young children is often difficult since physical characteristics are mild and not specific, and screening for ribosomal S6 kinase mutations is essential in most cases to confirm the diagnosis.^32^ In our case as well the diagnosis of Coffin–Lowry syndrome was considered only after discovering the deleterious mutation in *RPS6KA3*. This was a de novo somatic mutation not carried by the patient’s mother, a very common case in Coffin–Lowry disease.^27,33,34^ The mutation may explain some specific features of our patient (facial dysmorphism, toe and hand abnormalities, moderate cognitive and attention disabilities, and dilatation of Virchow–Robin spaces on MRI) but it is unlikely to contribute to pruritic ichthyosis, severe diffuse hypomyelination seen on MRI, and abnormal lipid peaks on ^1^H-MRS, features usually observed in SLS. Therefore, we attribute these features to the loss of ALDH1L2 protein. Such loss is defined by the nature of the compound mutations. Thus, the premature stop codon was predicted to produce a truncated protein (307 amino acid residues compared to 923 amino acid residues in normal protein), which includes 32 random amino acids at the C-terminus resulted from the frameshift. Such proteins are usually non-functional and are likely to undergo rapid degradation.^35^ In agreement with this conclusion, we did not detect the truncated protein in our patient’s fibroblasts. Of note, our previous studies indicated that the truncation of the enzyme beyond 290 aa (this does not include the mitochondrial leader sequence) produces non-functional protein.^13^ In the case of the frameshift mutation it would be 285 amino acids including the random peptide at the C-terminus. The splice site mutation in the second allele apparently compromised splicing, which would explain the drop in the mRNA level observed in patient’s fibroblasts. In fact, disrupted constitutive splicing most often results in loss of gene expression due to aberrant splicing.^14^

Our experiments with cultured patient’s fibroblasts provided a strong support for ALDH1L2 as a causative factor of patient’s conditions. Thus, cultured fibroblasts displayed abnormal mitochondrial morphology and very slow proliferation capacities, phenomena not reported for fibroblasts from patients with either Sjogren–Larsson or Coffin–Lowry syndrome. In fact, mitochondria in keratinocytes of SLS patients appear normal^36,37^ and our study demonstrated normal proliferation of fibroblasts from an SLS patient. Fibroblast cultures established from patients with Coffin–Lowry syndrome were characterized for signaling pathways downstream of RSK2^9,38,39^ but effects of the protein on proliferation or mitochondria function/morphology in such fibroblasts were not reported. Interestingly though, Rsk2 deficiency in mice was associated with enhanced proliferative capacity of fibroblast-like synoviocytes,^40^ the effect opposite to that observed for ALDH1L2-deficient fibroblasts in our experiments. Perhaps the strongest indication for ALDH1L2 mutations as the underlying cause of the neuro-ichthyotic syndrome in our patient was the restoration of the normal mitochondrial morphology and most of the metabotype, seen in father’s cells, after re-introduction of the wild-type enzyme to the patient’s fibroblasts.

*ALDH1L2* encodes a 923 amino acid residues (including 22 amino acids of the mitochondrial leader sequence in the N-terminus) protein, which resides in the mitochondrial matrix.^12^ So far deficiency of the *ALDH1L2* gene has not been reported, and the overall consequences of the enzyme loss for the cell are not yet clear. Nonetheless, the importance of mitochondrial folate pathways for the cell is well established,^41–44^ and recent reports further underscored the role of folate-bound mitochondrial serine metabolism for mitochondrial integrity and oxidative phosphorylation.^45–47^ Our study provides strong evidence that ALDH1L2 is a key player in these processes and the loss of the enzyme due to deleterious gene mutations leads to neuro-ichthyotic disease. What could be underlying mechanisms of such effect? ALDH1L2 enzyme converts 10-formyl-THF to THF and CO_2_ in an NADP^+^-dependent reaction thus producing NADPH (Fig. 2e).^15^ Though the biological role of this reaction is not fully understood, the enzyme could be a major source of NADPH in mitochondria.^19^ In turn, mitochondrial NADPH is crucial for the maintenance of reduced glutathione, the major antioxidant, and the loss of NADPH is associated with increased oxidative stress. In this regard, growing body of evidence indicates the link between oxidative stress and mitochondrial dysfunction.^48–50^ For example, increased oxidative stress in skin fibroblasts of patients with multiple acyl-CoA dehydrogenation deficiency led to fragmented mitochondria,^25^ the morphology similar to that observed in fibroblasts of our patient. In fact, the role of ALDH1L2 protein in preventing oxidative stress has been suggested by the study of melanoma cell metastasis.^51^ Thus, we propose that the ALDH1L2 loss induces mitochondrial dysfunction due to reduced NADPH and increased oxidative stress (Fig. 4g).

## Methods

### Legal authorization and ethics approval

The parents have given their permission (written consent form) for publication of the child’s photos. Written informed consents were obtained from parents for the genetic analyses. This report is in accordance with the French “Reference methodology” (MR-001) modified on 5th January 2006 and signed by the CHU of Clermont-Ferrand on 15th March 2007 for standard patient care. This research obtained authorization of the local ethics committee (CHU of Clermont-Ferrand).

### Reagents

All reagents were purchased from Sigma-Aldrich (St. Louis, MO, USA) unless otherwise specified.

### Gene sequencing

*ALDH3A2* exons, exon/intron junctions, and the full-length cDNA were sequenced essentially as we previously described.^52^ Whole exome sequencing was performed by IntegraGen SA (Evry, France) using the SureSelect V4 capture kit (Agilent, Massy, France) and the HighSeq2000 sequencer (Illumina, San Diego, CA)^53^ after written informed consent obtained from patient’s parents. Peripheral blood samples were drawn from the antecubital vein into 4 ml EDTA-containing tubes. Genomic DNA extraction was performed automatically from 2 ml of whole blood on a QIAsymphony SP instrument, by using the QIAsymphony DSP DNA Midi kit (QIAGEN), following the manufacturer’s protocol. There were 37,950 variations in the patient. Of these variants, 35,310 were classified as single nucleotide variations and 2640 were indels. Further analysis was focused on genes encoding proteins related to aldehyde dehydrogenases. This approach selected a single mutant gene. Interpretation was based on Human Genome Build 37 (NCBI/hg19).

### Generation of fibroblasts culture

Written consent has been obtained from parents prior to biopsies according to the institutional document for tissue biopsies and referring to the French laws for ethics and protection of subjects participating in medical research. Skin biopsies were performed at the anterior forearm after anesthesia with lidocaine/prilocaine cream applied topically for 1 h. A cylindrical skin plug including epidermis and dermis was removed using a sterile 3 mm skin punch and placed in 0.9% NaCl solution. Skin samples were washed and transferred to culture dishes containing DMEM/F12, 10% of FBS, penicillin/streptomycin/amphotericin (final concentration of 200 U/ml, 0.2 and 0.5 μg/ml, respectively) and maintained at 37 °C and under humidified air containing 5% CO_2_. After 1 week, the medium of the explant cultures was changed every 2–3 days. When confluent, cells were expanded. All fibroblast cell lines were maintained in DMEM (Gibco) supplemented with 10% FBS (Atlanta Biologicals, Flowery Branch, GA, USA) and 5% penicillin/streptomycin/neomycin cocktail (Gibco).

### Doubling time assay

Cell number was determined by hemocytometer or Countess II cell counter (Thermo, Waltham, MA, USA). In folate supplementation experiments, folinic acid or folic acid were added at 10 and 20 µM, respectively. Doubling time assays were performed by seeding a confluent 10-cm dishe of each cell line onto 6-cm dishes in triplicate. The seed time was considered *T*_0_ and cells were left to proliferate for 24 h. Cell numbers at 0 and 24 h were determined by trypan blue exclusion assay using hemocytometer.

### RNA extraction and cDNA synthesis

Total RNA was isolated from 2 × 10^6^ cells using an RNeasy mini kit (Qiagen). One microgram of total RNA was used in a reverse-transcription reaction to generate cDNA using high capacity cDNA reverse transcription kit (Applied Biosystems).

### Real-time PCR

Quantification of mRNAs was carried out by real-time PCR Realplex4 Mastercycler (Eppendorf, Hauppauge, NY, USA) using RT^2^ SYBR Green PCR master mix (Applied Biosystem) in final 20 μl PCR mixture containing 10 μl SYBR Premix EX Taq, 2 μl cDNA (100 ng), 0.4 μl (10 μM) forward and 0.4 μl (10 μM) reverse primers and 6.8 μl ddH_2_O. The PCR protocol was as follows: initial 95 °C melting for 5 min, then 40 cycles of denaturation at 95 °C for 30 s, annealing at 60 °C for 30 s, and elongation at 72 °C for 20 s. Levels of ALDH1L1 and ALDH1L2 mRNA were normalized by the levels of actin as housekeeping gene. The fold change in mRNA expression was calculated using 2^−ΔΔCt^.

### Cell lysate preparation and mitochondria isolation

Mitochondrial fractions were obtained using a mitochondrial isolation kit (MACS; Miltenyi Biotech, Auburn, CA, USA) following the manufacturer’s protocol.

### Real-time cell analysis

Experiments were carried out using an xCELLigence RTCA DP instrument (ACEA Biosciences, San Diego, CA, USA) placed in a humidified incubator at 37 °C and 5% CO_2_ according to the manufacturer’s manual. Cell proliferation was monitored using E-plate 16 (ACEA Biosciences). The background impedance reading for each well was set up using cell-free medium (100 µl per well) after pre-incubation at room temperature for 30 min. Cells were seeded in each well in 100 µl cell suspensions across a concentration range of 5 × 10^3^ to 8 × 10^4^ cells/well and allowed to attach for 30 min at room temperature. Plates were locked in the instrument, and impedance readings of each well were automatically recorded every 15 min for the duration of the experiment.

### Metabolomic analysis

Cells were cultured in 15 cm dishes, grown to 70–80% confluency, harvested, and subsequently flash frozen. Sample preparation for analysis was carried out at Metabolon Inc., as described.^54^ Briefly, individual samples were subjected to methanol extraction then split into aliquots for analysis by ultrahigh performance liquid chromatography/mass spectrometry (UHPLC/MS). The global biochemical profiling analysis comprised of four unique arms consisting of reverse phase chromatography positive ionization methods optimized for hydrophilic compounds (LC/MS Pos Polar) and hydrophobic compounds (LC/MS Pos Lipid), reverse phase chromatography with negative ionization conditions (LC/MS Neg), as well as a HILIC chromatography method coupled to negative (LC/MS Polar).^55^ All of the methods alternated between full scan MS and data dependent MS^*n*^ scans. The scan range varied slightly between methods but generally covered 70–1000 *m*/*z*. Metabolites were identified by automated comparison of the ion features in the experimental samples to a reference library of chemical standard entries that included retention time, molecular weight (*m*/*z*), preferred adducts, and in-source fragments as well as associated MS spectra and curated by visual inspection for quality control using software developed at Metabolon. Identification of known chemical entities was based on comparison to metabolomic library entries of purified standards.^56^

### Statistical analysis

Two types of statistical analyses were performed: (1) significance tests and (2) classification analysis. Standard statistical analyses were performed in ArrayStudio on log‐transformed data. For analyses not standard in ArrayStudio, the R program (http://cran.r-project.org/) was used. Following log transformation and imputation of missing values, if any, with the minimum observed value for each compound, Welch’s two sample *t*-test was used as significance test to identify biochemicals that differed significantly (*p* < 0.05) between experimental groups. An estimate of the false discovery rate (*q*‐value) was calculated to take into account the multiple comparisons that normally occur in metabolomic‐based studies. Classification analyses used included principal components analysis (PCA), hierarchical clustering, and OPLS-DA. For the scaled intensity graphics, each biochemical in original scale (raw area count) was rescaled to set the median across all samples equal to 1.

### ATP and NADPH/NADP^+^ assays

Cells were cultured in 15 cm dishes, harvested at 70–80% confluency, and flash frozen. ATP and NADPH/NADP^+^ were measured in whole cell lysate or isolated mitochondria using colorimetric ATP and fluorescence NADPH/NADP^+^ kits (Abcam), respectively according to the manufacturer’s protocols. Fifty microliters of the prepared sample were used in the assay. Experiments were carried out four times in triplicate.

### Metabolite extraction and HPLC assays

Metabolite extraction and HPLC analysis were performed according to published procedures.^57,58^ Cells cultured in 10-cm plates were washed two times with ice-cold PBS immediately prior to the addition of extraction buffer (1.5 ml of 9:1 methanol/chloroform mixture). Following the addition of extraction buffer, plates were scraped, and the extracts were kept at −20 °C overnight. Samples were spun down at 4 °C for 20 min at 21,000 × *g*, supernatants were placed to new Eppendorf tubes, and dried out using Centrivap (Labconco). Metabolites were dissolved in 1 mM Tris pH 9.0, passed through a 0.22 μm filter (Thermo), and resolved on a Symmetry C18 guard column (3.9 × 5 mm/4.6 × 250 mm, 5 μm particle size) using a Waters HPLC system (Milford, MA, USA). The elution solvents were (A) 0.1 M KH_2_PO_4_ pH 6.0 and (B) 0.1 M KH_2_PO_4_, 50% methanol pH 6.0. Elution conditions were solvent A (11 min) and a linear gradient of 0–20% solvent B. Peaks were detected by absorbance at 260 nm. The retention time was 8, 9, and 14 min for ATP, ADP, and AMP, respectively.

### Western blot analysis

Cells were lysed using RIPA buffer containing protease and phosphatase inhibitor cocktails. The Bradford protein assay (BioRad) was used to quantify protein concentrations. Equal amounts of protein (20 μg) were separated on a 7.5–12% Tris–glycine SDS polyacrylamide gel and were transferred to nitrocellulose membranes (GE). Membranes were blocked with 5% BSA in TBS supplemented with 0.1% Tween-20 for 1 h at room temperature and were incubated with primary antibody overnight at 4 °C. After incubating with horseradish peroxidase-conjugated secondary antibodies (anti-rabbit, NXA931; anti-mouse, NA934V; both from GE; 1:10,000), membranes were developed using supersignal chemiluminescence reagents (Thermo). Blots were striped with a buffer containing 1.5% glycine, 0.1% SDS, and 1% Tween-20 at pH 2.2. ALDH1L2 was detected using in-house ALDH1L2-specific polyclonal antibody (1:2000).^12^ ALDH3A2 (ab113111), VDAC (ab154856), and GAPDH (ab8245) antibodies were from Abcam. Actin antibody (sc-47778) was purchased from Santa Cruz. All commercial antibodies were used at the dilution of 1:1000. All blots derived from the same experiment were processed in parallel. Band intensities were quantified using ImageJ software, NIH (https://imagej.nih.gov/ij/). Uncropped blot images are shown in Supplementary Fig. 12.

### Immunocytochemistry

Cells were plated onto glass bottom microwell dishes (MatTek Corp., Ashland, MA, USA), left to attach overnight and then were stained with 250 nM TMRM, 100 nM Mitotracker, and 0.5 μg/ml Hoechst (all from Molecular Probes). In a separate experiment, cells were also stained with 40 μM H_2_DCFDA for whole cell ROS visualization. Cells were stained independently and imaged immediately following dye incubation to ensure changes in ROS were not a product of imaging time differences. For fixed cell immunocytochemistry, cells were cultured on glass coverslips and subsequently fixed in 4% paraformaldehyde, quenched with ammonia sulfate, and washed with PHEM buffer. Cells were permeabilized with 0.1% Triton-100X and blocked with 5% goat serum for 1 h at room temperature. Cells were then stained with anti-ALDH1L2 antibody (1:250) for 1 h, rinsed with PHEM, and incubated with secondary anti-rabbit IGG goat antibody (Molecular Probes, A21441, 1:500). Coverslips were mounted to glass slides with mountant containing DAPI (Thermo) and left to dry overnight at 4 °C and were imaged the following day.

### Analysis of mitochondrial mass and membrane potential

Cells (1.5 × 10^5^) were seeded onto 6 cm plates and left to attach overnight. Cells were harvested, resuspended in 5 ml of fresh medium and stained with 250 nM TMRM and then with 100 nM MitoTracker Green (both from Molecular Probes) to determine mitochondrial polarization and mitochondrial mass respectively. Intensity of each dye was determined using CytoFLEX flow cytometer (Beckman Coulter, Indianapolis, IN, USA). Quantitative analysis was completed using CytoFLEX software.

### ALDH1L2 lentiviral transduction

The construct of human ALDH1L2 cDNA cloned to a pLenti-GIII-CMV lentiviral vector was purchased from Applied Biological Materials Inc. (Richmond, BC, Canada). Recombinant lentivirus for the ALDH1L2 expression was produced using ViraPower lentiviral expression system (Thermo) according to the manufacturer’s protocol. Patient’s fibroblasts grown in a 6-well plate (1 × 10^6^ cells per well) were transduced with 2 ml of the mixture of RPMI and the generated viral stock (1:1) for 24 h. The efficiency of lentiviral transduction was confirmed by the killing curve selection assay with puromycin and individual clones were cultured.

### Assays of reduced folate pools

Approximately 5 × 10^6^ cells were collected and rapidly washed three times with ice-cold PBS. The cell pellet was resuspended in 50 mM Tris–HCl buffer, pH 7.4, containing 50 mM sodium ascorbate. Cells were lysed by heating for 3 min in a boiling water bath. Cell lysates were chilled on ice and centrifuged for 5 min at 17,000 × *g* at 4 °C. Folate pools were measured in cell lysates by the ternary complex assay method as described.^59,60^ Folate levels were calculated per mg of cellular protein measured by Bradford assay.

### Transmission electron microscopy

Cells were plated on Nunc Permanox slide chambers (3000 cells per well) in DMEM and left to attach overnight. Cells were fixed for 1 h at room temperature with 2.5% formaldehyde/glutaraldehyde (1:1) in 0.1 M sodium cacodylate buffer pH 7.4. Slides were stored at 4 °C until further processing. Following three rinses with 0.1 M sodium cacodylate buffer, pH 7.4, cells were post-fixed with 1% osmium tetroxide/1.25% potassium ferrocyanide/0.1 M sodium cacodylate buffer for 1 h at room temperature. After washes in deionized water, cells were dehydrated using increasing concentrations of ethanol (30%, 50%, 75%, and 100% twice, 10 min each) and embedded in Polybed 812 epoxy resin (Polysciences, Inc., Warrington, PA). Cells were sectioned en face to the substrate at 70 nm using a diamond knife and Leica Ultracut UCT ultramicrotome (Leica Microsystems, Inc., Buffalo Grove, IL). Ultrathin sections were collected on 200 mesh copper grids and stained with 4% aqueous uranyl acetate for 12 min, followed by Reynolds’ lead citrate for 8 min. Samples were observed with a JEOL JEM-1230 transmission electron microscope operating at 80 kV (JEOL USA, Peabody, MA) and digital images acquired using a Gatan Orius SC1000 CCD camera and Gatan Microscopy Suite 3.0 software (Gatan, Inc., Pleasanton, CA). Magnification for each image set includes 5000×, 10,000×, and 20,000×.

### Reporting summary

Further information on research design is available in the Nature Research Reporting Summary linked to this article.

## Supplementary information

Supplementary data file 1

Supplementary Figures

Reporting Summary

## Data Availability

All data generated or analyzed during this study are included in this published article and Supplementary Information files or are available from the author upon reasonable request.

## Supplementary information

**Supplementary information** accompanies the paper on the *npj Genomic Medicine* website (10.1038/s41525-019-0092-9).
