# Supplementary material for: Deleterious mutations in ALDH1L2 suggest a novel cause for neuro-ichthyotic syndrome
Source: NPJ Genom Med. 2019 Jul 23;4:17. doi: 10.1038/s41525-019-0092-9 (PMC6650503; doi:10.1038/s41525-019-0092-9)
Supplement: Supplementary file 2 — Supplementary Figures [file 41525_2019_92_MOESM2_ESM.pdf]

SUPPLEMENT (Sarret et al)

1. Supplementary figures 1-12

2. Supplementary table 1 (separate Excel file)

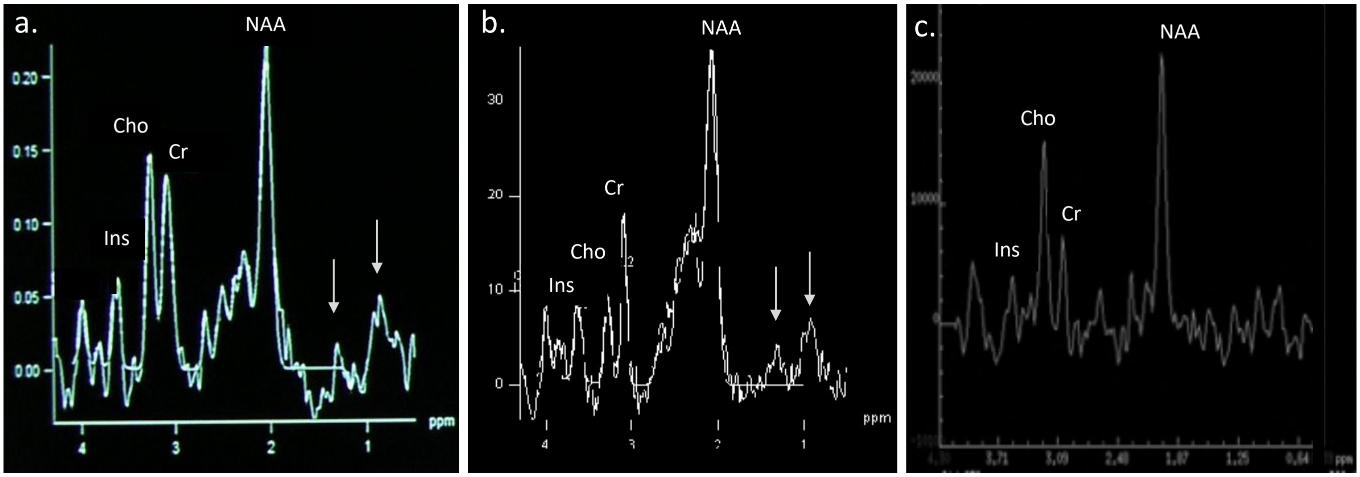

Supplementary Fig. 1. Evolution of the spectrum of ^{1}H-MRS in the corona radiata for our patient shows a major lipid peak at 0.9 ppm and a smaller lipid peak at 1.3 ppm (*arrows*) at the age of 2 years (**a**) and 6 years (**b**). These peaks were not clearly visualized at the age of 14 years (**c**). NAA, N-acetylaspartate; Cr, creatine; Cho, choline; Ins, inositol; ppm : particles per million.

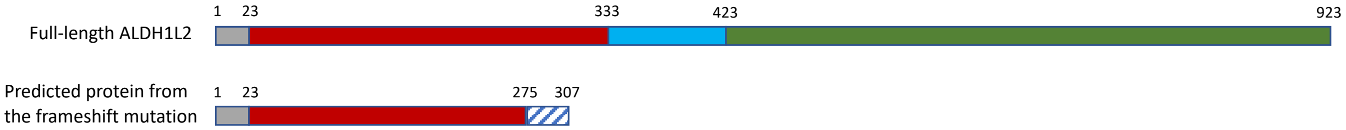

Supplementary Fig. 2 Full-length ALDH1L2 is a 923 aa protein consisting of the mitochondrial leader sequence (aa 1-22, *grey*); the N-terminal folate-binding domain (aa 23-333, *red*); the intermediate formyl carrier domain (aa 334-423, *blue*); and the C-terminal aldehyde dehydrogenase domain (aa 424-923, *green*).[1] The frameshift mutation c.827del/p.Val276Glyfs*33 creates a premature stop codon leading to a truncated protein product, which has a random peptide sequence (*shaded box*) due to the frame shift. Predicted protein sequence was derived from mutant ALDH1L2 cDNA analyzed using on line ExPASy translate tool (https://web.expasy.org/translate/).

1. Krupenko, N.I. *et al.* ALDH1L2 is the mitochondrial homolog of 10-formyltetrahydrofolate dehydrogenase. *J. Biol. Chem.* **285**, 23056-63 (2010).

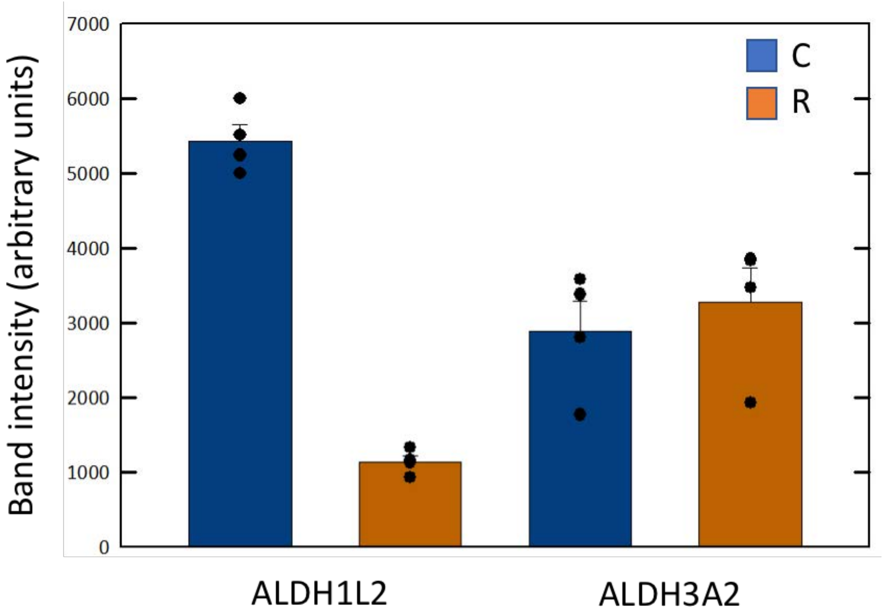

Supplementary Fig. 3. Levels of ALDH1L2 and ALDH3A2 proteins in C (control) and R (patient) fibroblasts calculated from Fig. 2a as relative band intensities. Mean ± SE are shown. P value for ALDH1L2 was below 0.0001 (Student's t-test).

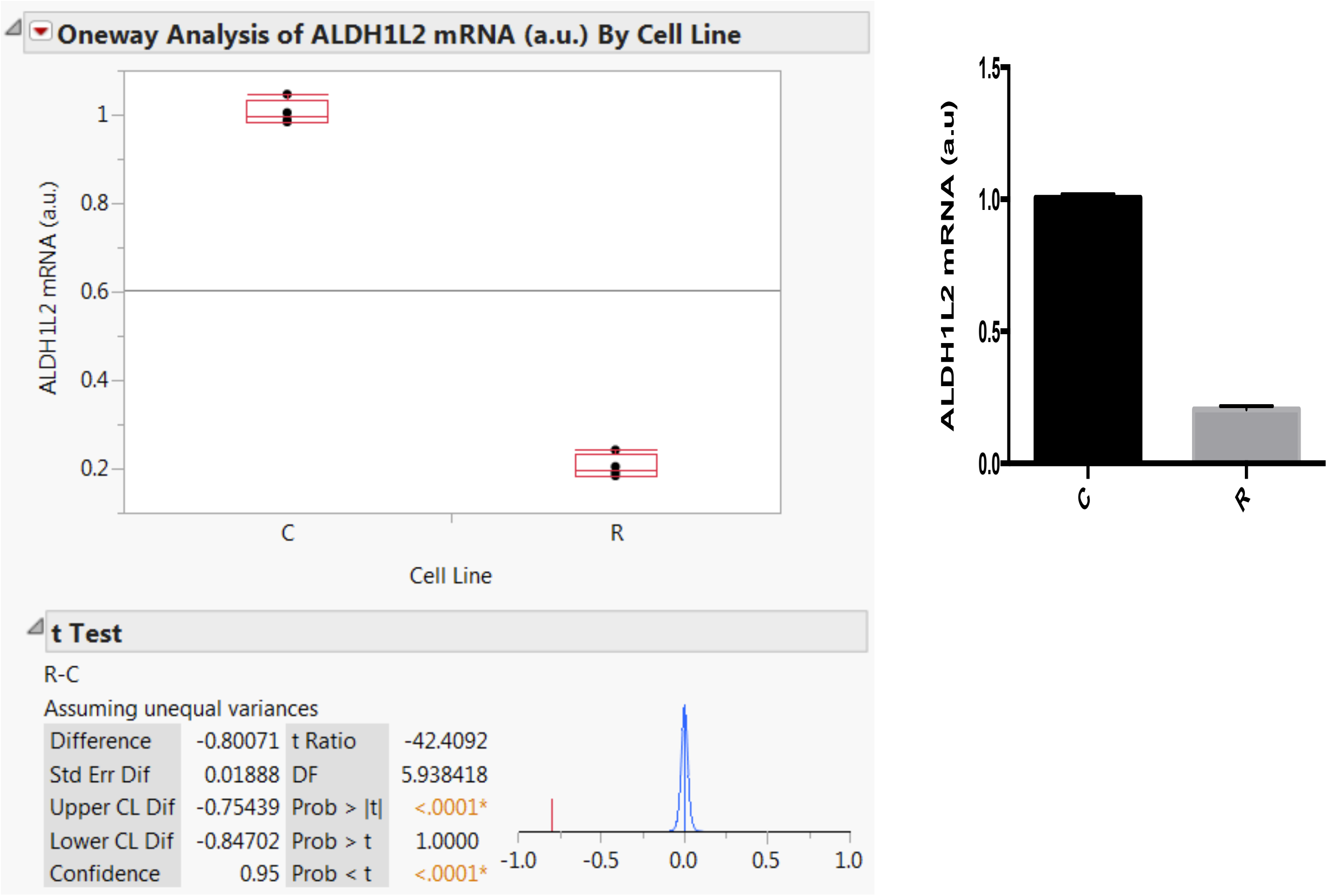

Supplementary Fig. 4. Levels of mRNA in fibroblasts of SLS-like patient (*R*) and healthy individual (control, *C*) measured by real-time PCR. For each cell type, mRNA was isolated from three samples each collected from a different plate (biological replicates). Each sample was analyzed 4 times (technical replicate). Averages of technical replicates were used to calculate mean ± SE.

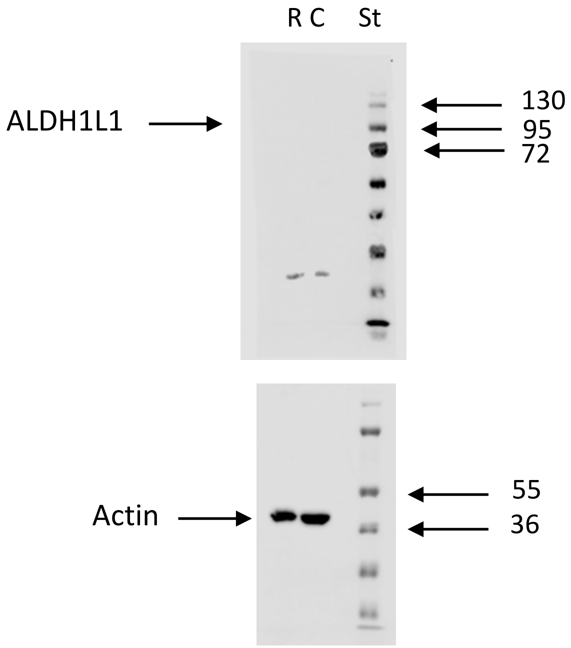

Supplementary Fig. 5. Fibroblasts from SLS-like patient (R) and healthy individual (control, C) lack ALDH1L1 (cytosolic 10-formyltetrahydrofolate dehydrogenase) expression. *Upper panel*, Western blot assay of ALDH1L1 using specific polyclonal antibody; *lower panel*, actin shown as the loading control.

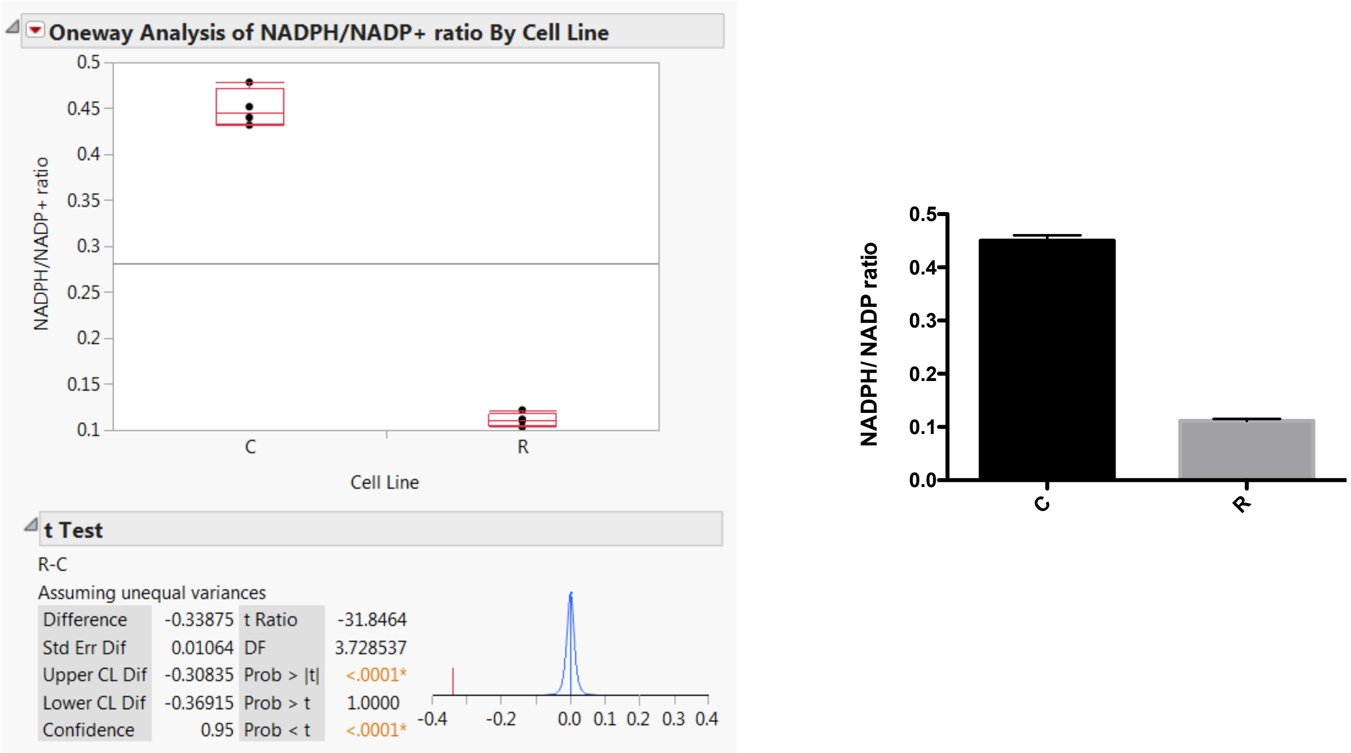

Supplementary Fig. 6. NADPH/NADP$^+$ ratio in fibroblasts of SLS-like patient (*R*) and healthy individual (control, *C*) measured by a fluorescence kit. Four samples (biological replicates) were analyzed for each cell type. Each sample was measured 4 times (technical replicate). Averages of technical replicates were used to calculate mean ± SE.

ATP assay in whole lysate(C,R)

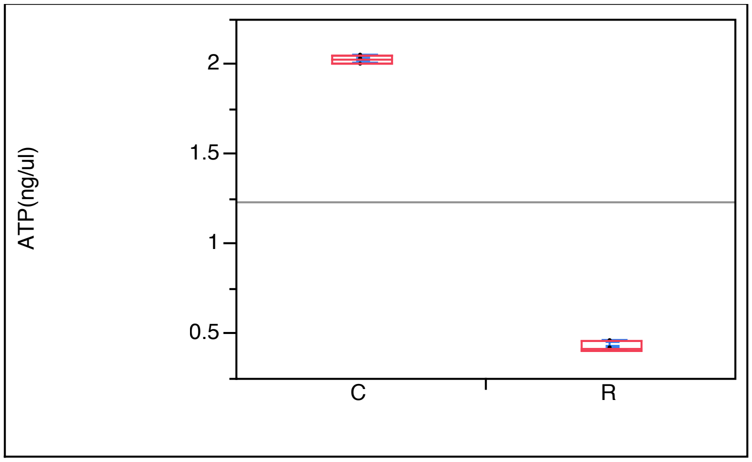

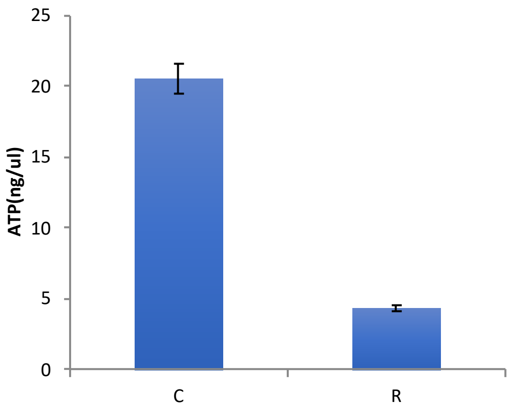

Means and Std Deviations

Level	Number	Mean	Std Dev	Std Err Mean	Lower 95%	Upper 95%
C	3	2.02667	0.025166	0.01453	1.9642	2.0892
R	3	0.42900	0.027622	0.01595	0.3604	0.4976

t Test

R-C

Assuming unequal variances

Difference	-1.5977	t Ratio	-74.0546
Std Err Dif	0.0216	DF	3.965801
Upper CL Dif	-1.5376	Prob > |t|	<.0001*
Lower CL Dif	-1.6578	Prob > t	1.0000
Confidence	0.95	Prob < t	<.0001*

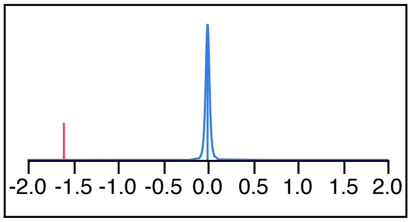

Supplementary Fig. 7. Levels of ATP in fibroblasts of SLS-like patient (*R*) and healthy individual (control, *C*) measured by a colorimetric assay. Three samples (biological replicates) were analyzed for each cell type. Each sample was measured 4 times (technical replicate). Averages of technical replicates were used to calculate mean ± SE.

ATP assay in mitochondria fraction (C, R)

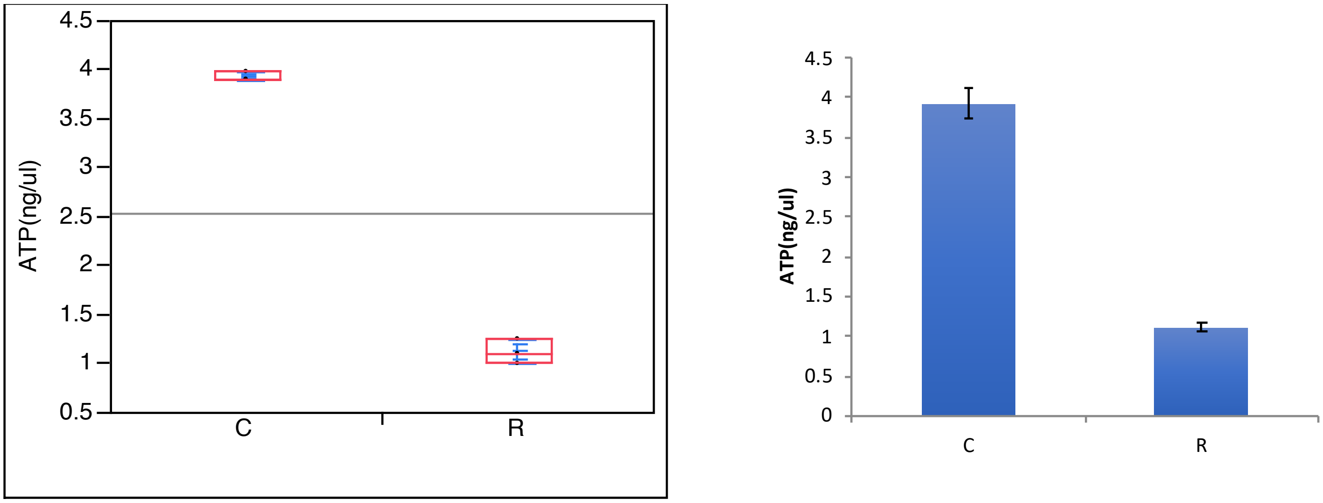

Means and Std Deviations

Level	Number	Mean	Std Dev	Std Err Mean	Lower 95%	Upper 95%
C	3	3.92667	0.046188	0.02667	3.8119	4.0414
R	3	1.11667	0.125831	0.07265	0.8041	1.4292

t Test

R-C

Assuming unequal variances

Difference	-2.8100	t Ratio	-36.3106
Std Err Dif	0.0774	DF	2.529338
Upper CL Dif	-2.5356	Prob > ltl	0.0002*
Lower CL Dif	-3.0844	Prob > t	0.9999
Confidence	0.95	Prob < t	<.0001*

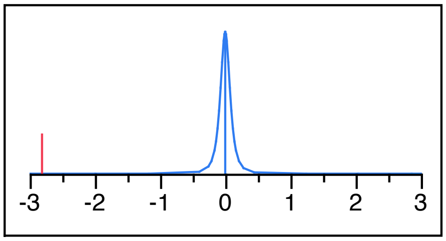

Supplementary Fig. 8. Levels of ATP in mitochondria isolated from fibroblasts of SLS-like patient (*R*) and healthy individual (control, *C*) measured by a colorimetric assay. Three samples (biological replicates) were analyzed for each cell type. Each sample was measured 4 times (technical replicate). Averages of technical replicates were used to calculate mean ± SE.

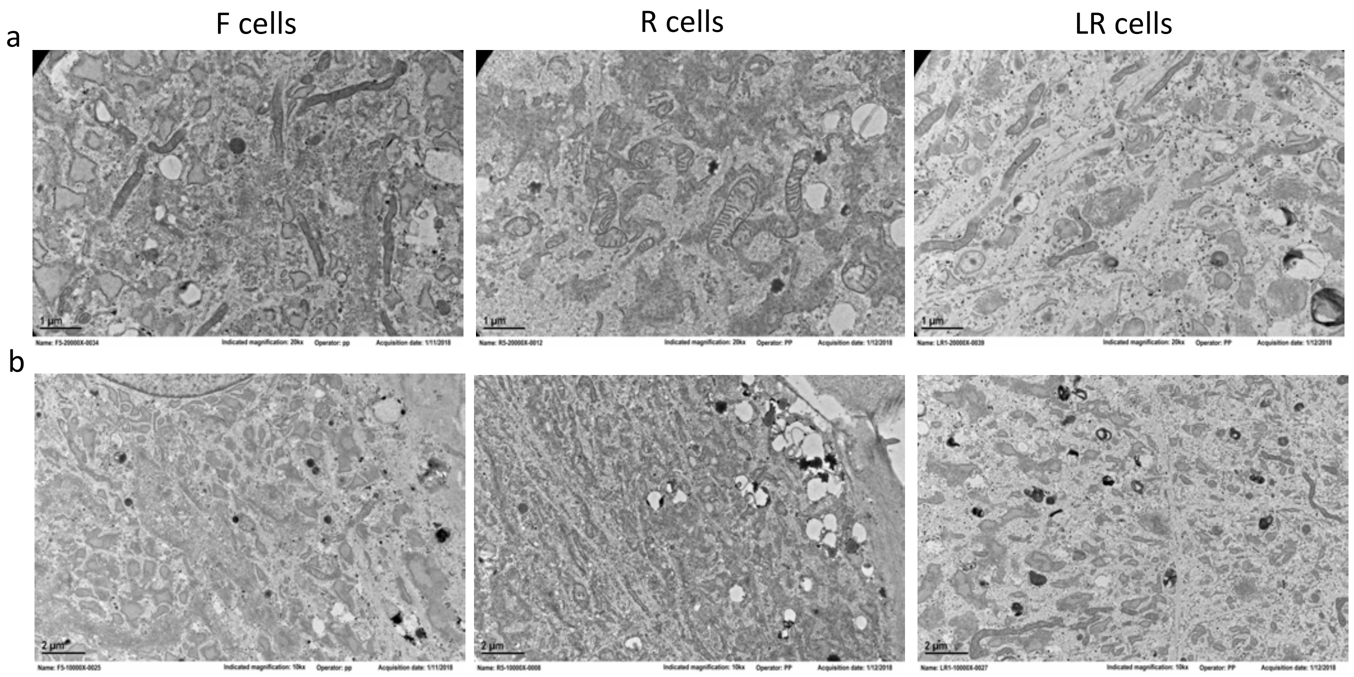

Supplementary Fig. 9. Transmission electron microscopy of R, F, and LR cells. Cells were plated in Permanox slide chambers at 3000 cells/well in DMEM and left to attach overnight. Cells were fixed and processed as described in Materials and Methods. **a**, Electron microscopy images taken at 20000x magnification; difference in the mitochondrial morphology are seen between R and F/LR cells). **b**, Electron microscopy images taken at 10000x magnification; large vesicles are seen in R but not in F/LR cells.

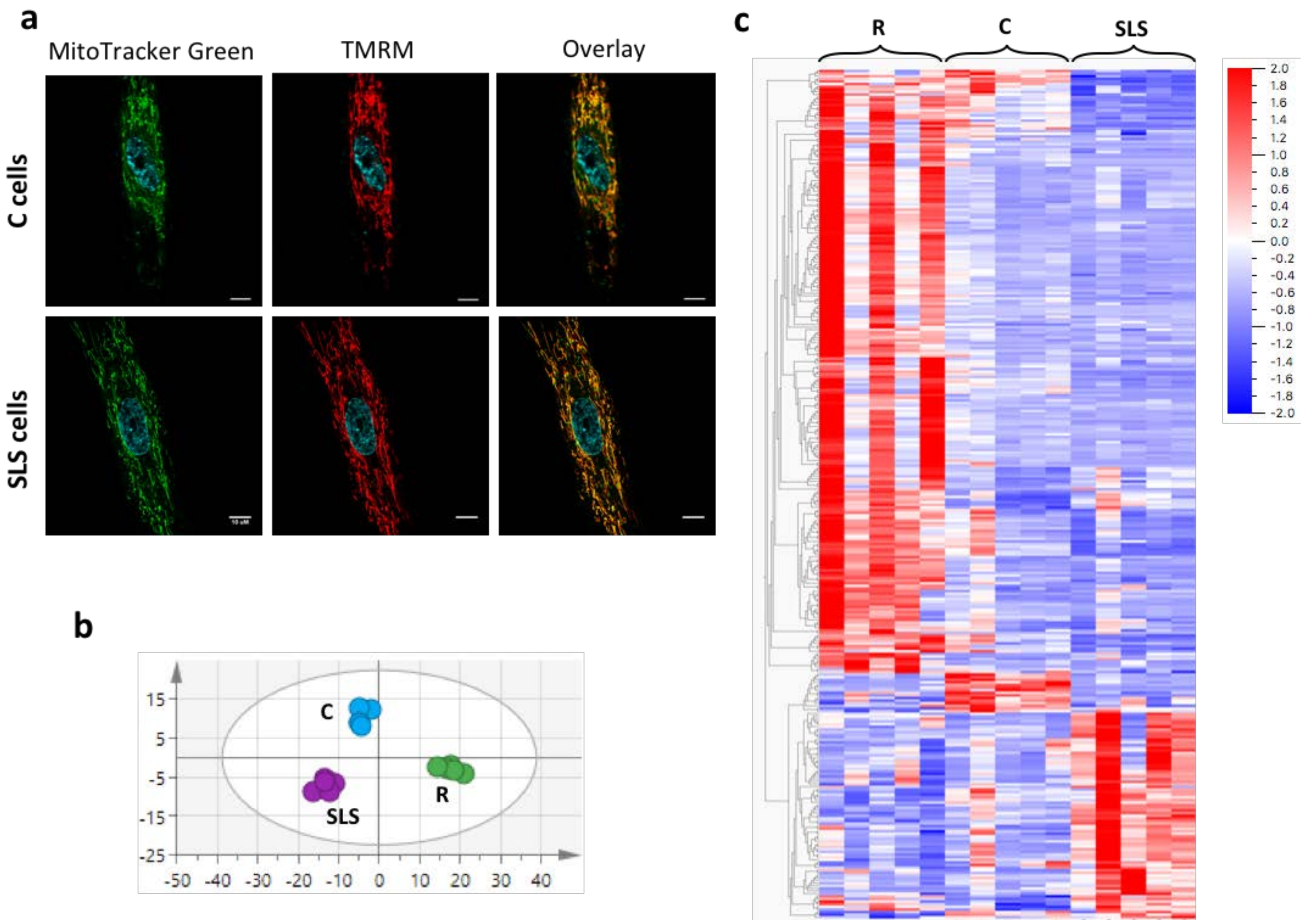

Supplementary Fig. 10. a, Mitochondria from fibroblasts of SLS patient and a healthy individual have similar morphology. **b,** OPLS-DA analysis (performed with SIMCA Version 15.0.2, Sartorius Stedim Data Analytics AB, Umeå, Sweden) of metabolomic data for R, C and SLS fibroblasts (total of 516 metabolites were analyzed). **c,** Heat map representation of the metabolite comparison between R, C and SLS cells (n=5, data were filtered by *p* value ≤ 0.05). Data analysis was performed using Qlucore Omics Explorer v.3.4 software, Qlucore, Lund, Sweden.

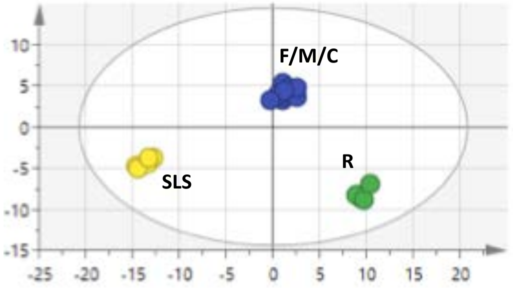

Supplementary Fig. 11. OPLS-DA analysis of metabolomic data. Fibroblasts from SLS patient (SLS), our patient with SLS-like symptoms (R), this patient parents (F, father; M, mother), and another healthy individual (C) were analyzed. All asymptomatic individuals (patient parents and a healthy individual with no connection to the family) were analyzed as a single group. Analysis was performed using SIMCA Version 15.0.2, Sartorius Stedim Data Analytics AB, Umeå, Sweden.

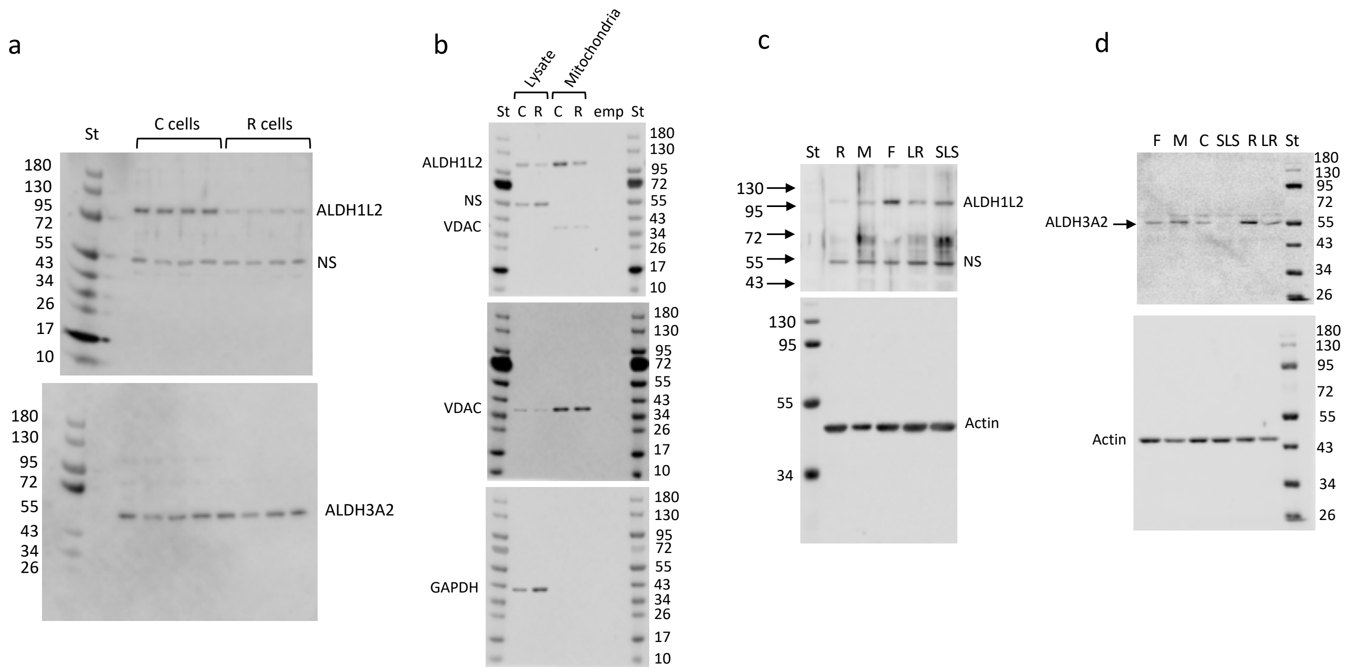

Supplementary Fig. 12. Uncropped western blots. a, Full blots for Fig. 2a. **b**, Full blots for Fig. 2c. **c, d,** Full blots for Fig. 3a. All blots in each panel were derived from the same experiment and were processed in parallel. Twenty µg of protein was loaded in each lane. NS, non-specific band seen in whole cell lysates with ALDH1L2 antibody.
